# Demographics of the semi-slug *Parmarion martensi*, an intermediate host for *Angiostrongylus cantonensis* in Hawai‘i, during laboratory rearing

**DOI:** 10.1017/S0031182020001353

**Published:** 2021-02

**Authors:** Lindsey J. Hamilton, Yaeko Tagami, Lisa Kaluna, John Jacob, Susan I. Jarvi, Peter Follett

**Affiliations:** 1 USDA Daniel K. Inouye Pacific Basin Agricultural Research Center, 64 Nowelo St, Hilo, HI 96720, USA; 2 University of Hawai‘i at Hilo, College of Pharmacy, 722 South Aohoku Place, Hilo, HI 96720, USA

**Keywords:** *Angiostrongylus cantonensis*, captive rearing, life-history, *Parmarion martensi*, rat lungworm, semi-slug, slug vector

## Abstract

The semi-slug, *Parmarion martensi*, is an intermediate host of the zoonotic nematode, *Angiostrongylus cantonensis*, the aetiological agent of neuroangiostrongyliasis or rat lungworm disease in humans. Rearing methods were developed for *P. martensi* to facilitate studies on nematode transmission and control. *Parmarion martensi* exhibited high survivorship when reared on a diet of dog food and fresh fruits and vegetables in temperature-controlled cabinets at 21.4°C, 98% relative humidity and 12:12 L:D cycle. Rearing containers were lined with moist paper towels for substrate and plastic pots were provided for hiding/resting and egg-laying. Under these conditions, time to first reproduction was 165.3 ± 12.3 days, fecundity was approximately 34.5 ± 7.8 eggs per adult, and hatch rate was 52.7 ± 3.2%. Survivorship post egg hatch was 86.2 ± 2.9% at 30 days (neonates had a mortality rate of about 14%) and 99% thereafter for up to a year. The demographics of laboratory-reared and wild-caught *P*. *martensi* were similar except for the weight of reproductive adults, which was significantly higher in laboratory-reared adults (4.0 ± 0.2 g) than in field-collected adults (1.5 ± 0.1 g).

## Introduction

*Angiostrongylus cantonensis*, the rat lungworm, is a zoonotic, parasitic nematode responsible for neuroangiostrongyliasis or rat lungworm disease and the leading cause of human eosinophilic meningitis worldwide (Hochberg *et al*., 2007; Graeff-Teixeira *et al*., 2009; Prociv and Turner, 2018). Rats serve as the definitive hosts of *A. cantonensis*, and gastropods (snails and slugs) are the obligatory intermediate hosts and may all be presumed capable of being infected unless proven otherwise. *Angiostrongylus cantonensis* development occurs from the first larval stage (L1) through the third larval stage (L3) in the gastropod intermediate host and the L3 stage is the only stage known to infect vertebrate hosts, including the definitive rat hosts and accidental hosts such as humans (Cowie, 2013). Transmission to humans is thought to be due to intentional or accidental ingestion of L3 larvae in infected gastropod tissue (Medeiros *et al*., 2020). *Angiostrongylus cantonensis* is prevalent in Southeast Asia and tropical Pacific islands. It has also been detected on the continental United States in intermediate and/or non-human definitive or accidental hosts in Louisiana, Florida, Oklahoma, Alabama and Mississippi, and human cases have been reported in Louisiana, Texas and Tennessee (Barratt *et al*., 2016; Flerlage *et al*., 2017; Stockdale Walden *et al*., 2017).

**Fig. 1.**
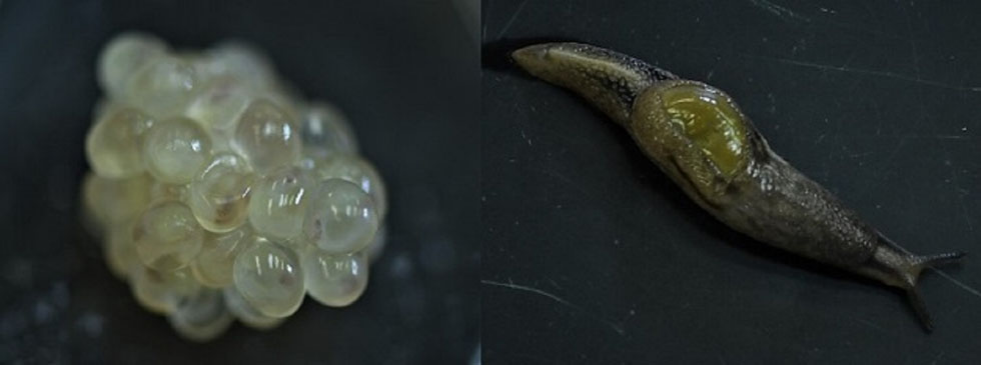
*Parmarion martensi* egg cluster (left) at 1 day before emergence, showing developing neonates, and a mature adult (right).

In Hawai‘i, the invasive semi-slug, *Parmarion martensi* (Fig. 1), has become the most competent intermediate host with both high infection prevalence and high parasite loads compared to most other species of terrestrial gastropods (Hollingsworth *et al*., 2007; Qvarnstrom *et al*., 2010, 2013; Kim *et al*., 2014; Medeiros *et al*., 2020). Due to high infection prevalence and load, and its common association with human habitations, *P. martensi* is suspected to be one of the main drivers of human infection, particularly in east Hawai‘i Island (Hollingsworth *et al*., 2007; Jarvi *et al*., 2018). Outbreaks of human infection may have been associated with the invasion of *P. martensi* on Hawai‘i Island, Maui and Okinawa, Japan (Asato *et al*., 2004; Hollingsworth *et al*., 2007; Jarvi *et al*., 2020).

*Parmarion martensi* has not yet been detected on the U.S. mainland. Due to the potential severity of the disease in Hawai‘i and elsewhere, the possibility of increased risk factors for exposure due to parasite and/or host population expansion, and a shortage of definitive treatment options, the prevention of neuroangiostrongyliasis is of primary concern. The California Department of Agriculture intercepted *P. martensi* 37 times between 2009 and 2016 and is now using dogs to detect this quarantine species on nursery stock and plant parts from Hawai‘i (Leathers, 2016).

*Parmarion martensi* is hermaphroditic and may function as both male and female. In Taiwan, *P. martensi* was successfully reared in the laboratory on cabbage, both singly and in pairs (Liu *et al*., 1997). Standardized rearing methods for *P. martensi* would facilitate the development of information on biotic and abiotic factors related to vector competence and transmission, and evaluation of management tactics. Recent studies in Hawai‘i have used field-collected *P. martensi* as a source of *A. cantonensis* for experimentation (Jarvi *et al*., 2012, 2015, 2019; Howe *et al*., 2019); however, wild-caught individuals may also be carrying other nematode species (Howe *et al*., 2019). Evaluation of management tactics relies on performance comparisons between treated and untreated control groups, which can only be accomplished with a reliable rearing system. Here we describe laboratory rearing methods that provide high survivorship, longevity and sustained reproduction in *P. martensi*.

## Materials and methods

To initiate a laboratory colony of *P. martensi*, wild adults were hand-collected from around buildings and off plants near residential areas after sunset in the Hilo and Puna areas of eastern Hawai‘i Island (GPS coordinates 19.700066, −155.082889; 19.581760, −154.969746). Traps were also deployed, consisting of folded plastic sheets with water and dog food placed between the folds to increase the number of semi-slugs captured. We collected adult *P. martensi* in March, May and September 2019 consisting of 12, 6 and 2 individuals, respectively. They were assumed to be at reproductive age and weighed between 0.47 and 2.16 g (mean = 1.53 ± 0.1 g). These adults were held in rearing containers singly (4) or in pairs (8).

### Juvenile and adult rearing

Laboratory rearing was conducted at the Waiākea Experimental Station of the University of Hawai‘i at Hilo, Hilo, Hawai‘i. During the initial development of *P. martensi* rearing methods, air temperature in the rearing cabinets was poorly controlled and mean temperatures fluctuated between 21.1 and 29.6°C (mean = 24.5). Under these temperatures, laboratory-reared adult *P. martensi* survived but laid unviable eggs. Wild-collected adults held under these conditions laid eggs that developed and hatched successfully, but all neonates died within the first month. New temperature control equipment was installed, and after extensive trial and error, the optimal rearing temperature for *P. martensi* was determined to be approximately 21–22°C based on the survival of viable eggs into adults. All further studies were conducted at these lower temperatures (range 21.1–21.7°C; mean = 21.4) and are the conditions under which all the results reported here were produced.

**Fig. 2.**
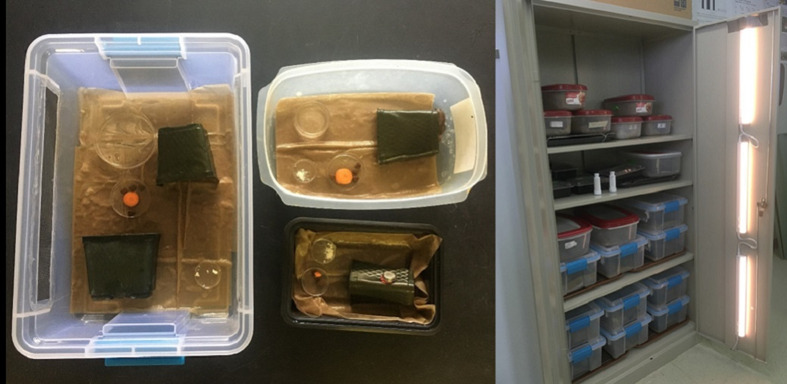
*Parmarion martensi* rearing containers (left) with moist paper towels, food and shelter for hiding and egg laying, and rearing cabinet (right) with a modified lighting system.

Different life stages were held in different types of containers. Juveniles in were held in 20.3 × 12.7 × 5.1 cm^3^ plastic containers (150 Black Tripak 6350, TRIPAK Industrial USA, LLC) whereas adults were held in larger containers of dimensions 29.2 × 19.1 × 10.2 cm^3^ (RUBBERMAID FLEX & Seal N Saver 4.2 Litre, Newell Brands Inc.) or 35.6 × 25.4 × 16.5 cm^3^ (Sterilite 12 qt Gasket Box, Sterilite^®^). Garden soil, coir and paper towels were tested for their suitability as substrate. Unbleached paper towels (Brown M-Fold Paper Towels, 9.2 × 9.4, Pacific Blue Basic™) saturated with distilled water were determined to be the best substrate because they were inexpensive, easy to replace and maintained high humidity while providing high *P. martensi* survivorship in the ventilated containers. Variously sourced square-form plastic pots (10 × 10 cm^2^) were cut into halves or quarters and placed in the containers (Fig. 2) to provide suitable hiding, resting and egg-laying habitat for *P. martensi*. A 60–90 cm diameter Petri dish full of water was also provided to help maintain high relative humidity and as an additional source of water. All containers with juveniles and adults were held in modified metal wall cabinets under a 12:12 light cycle using 500 lumens LED lights, set at 50% brightness, and 2700 K warm white (Fig. 2). Environmental conditions in the rearing containers in the cabinets were monitored using temperature/relative humidity data loggers (Thermocrons iButton type DS1923; Maxim/Dallas Semiconductor Corp., USA) recording hourly. At 21–22°C, relative humidity within the smaller and less ventilated juvenile containers ranged from 99.2 to 100% (mean = 99.7) and the larger adult containers ranged from 77 to 100% (mean = 98).

Juveniles or adults within each rearing container were fed and cleaned every 2–3 days. Fresh food consisted of a continuous supply of dry kibble dog food (for protein), crushed cuttlebone (a calcium supplement) and a piece of fresh produce such as papaya, avocado, cucumber, potato or dried oats. Food was provided in sufficient amounts to ensure that they did not deplete it between feedings. Feces were removed regularly by wiping container walls and plastic pots with a Kimwipe™ or paper towel. Juvenile *P. martensi* were transferred into the larger adult rearing containers when they reached approximately 30 days old.

Once standard rearing methods were established, *P. martensi* were reared with siblings from a common egg cluster in cohorts of 2–15 individuals. Under these rearing conditions, data were taken on growth and development including weight, survivorship, time to first egg cluster (first hatch – first egg cluster collected), the number of eggs per egg cluster, egg development period (egg cluster collection – first egg hatch) and egg hatch rate (total successfully hatched neonates per cluster/total eggs counted per cluster). Reproductive statistics were averaged for a cohort assuming equal contribution as egg clusters could not be assigned to individual adults. Data on egg production and juvenile and adult survivorship were taken every 2–3 days, whereas body weights were measured every 2–4 weeks. If an individual appeared to be in poor health, it was immediately isolated in a separate container.

Small paintbrushes and feather forceps were used to manipulate individuals for data collection. All instruments used for rearing were disinfected with 70% isopropyl or 70% ethyl alcohol between servicing containers to prevent transmission of potential contaminants. Rearing containers and their contents were sanitized bi-weekly by soaking for 20 min in a 10% bleach solution and replacing the substrate.

### Egg care

*Parmarion martensi* adults laid eggs in clusters, typically attached to the underside of the plastic pots, or occasionally on the lid of the rearing container. Egg clusters were three-dimensional, with eggs laid on top of other eggs in a mass. Newly laid egg clusters were weighed, and the number of eggs was estimated by visual counting of the intact egg cluster. Individual egg clusters were placed in 60 mm Petri dishes with a damp Kimwipe™, sealed with parafilm, and stored under 24 h darkness. The Petri dishes were opened every 2–3 days for gas exchange. At first hatch, a piece of dog food and cuttlebone were placed near the egg cluster. Egg hatching was deemed completed when there were no unhatched developed eggs visible. Unhatched developed eggs were easily discernible from unviable eggs by the appearance of the developing neonate inside the clear eggshell (Fig. 1). At this point, the Petri dish was opened and set inside a standard plastic rearing container and once the neonates had dispersed into the container, the Petri dish was removed. For each egg cluster, the egg-laying date, weight, first and last hatch dates, percent hatched and the number of successfully hatched neonates that survived for 30 days were recorded. A chi-square test of independence was performed to examine egg production, hatch rate and survivorship between egg clusters produced by laboratory-reared and wild-collected adults. A Wilcoxon rank-sum test was performed to compare the weights of reproductive laboratory-reared and wild-collected adults. All statistical tests were performed with R v. 3.6.3.

## Results

### Reproduction

From our initial collection of wild adults, only one of the individuals held alone produced eggs and none hatched. For paired adults, a total of 461 eggs were laid between 3 June and 25 October 2019, and 298 neonates hatched from the eggs. From these F1 neonates, a subset of 78 individuals, composed of nine cohorts of 2–15 (mean = 8.5) were followed for 280–334 days of age to document reproduction. All 78 adults were alive, and some were still reproducing when the study was ended because of time constraints. Although egg production had slowed to <1 egg cluster per month for all groups, the resulting data on time from first to last egg cluster produced and total fecundity is probably underestimated. The time to first reproduction varied from 130 to 240 days, with a mean = 165.3 ± 12.3 (median = 152.0) days, and the time from first to last egg cluster produced was 32–161 days (mean = 100.8 ± 15.6, median = 89.0). Eggs were produced in clusters of 2–84 eggs (mean = 27.4 ± 1.3) that weighed 0.02–1.79 g (mean = 0.67 ± 0.03). Fecundity per adult was 0.5–68.0 eggs (mean = 34.5 ± 7.8).

Over the course of the laboratory-rearing effort, 112 egg clusters were collected from both *P. martensi* captured in the wild and their F1, laboratory-reared offspring. All egg clusters were monitored for development, hatch and 30-day survival of successfully hatched neonates (Table 1). Egg clusters produced by laboratory-reared and wild-collected *P. martensi* adults were not significantly different for any of these measurements (*χ*^2^_(3, *N*_ _=_ _479)_ = 0.47481, *P* = 0.92).

**Table 1.** *Parmarion martensi* egg cluster statistics: egg development (egg cluster collection to first observed hatch); hatch duration (first observed hatch to absence of visible unhatched developed eggs); hatch rate (estimated number of eggs per cluster/number hatched per cluster); 30-day survival (number of successfully hatched neonates per cluster that survived the first 30 days) are presented as percentages.

	Egg clusters									Neonates				
	Egg development (days)			Hatch duration (days)			Hatch rate (%)			30 day survival (%)				
	*n*	*M* ± s.e.	Range	*n*	*M* ± s.e.	Range	*n*	*M* ± s.e.	Range		*n*	*M* ± s.e.	Range	
All	112	15.0 ± 0.2	6–24	117	3.5 ± 0.2	1–14	161	52.7 ± 3.2	0–100		89	86.2 ± 2.9	0–100	
Captive^a^	91	15.1 ± 0.2	11–21	95	3.4 ± 0.2	1–14	127	54.7 ± 3.5	0–100		70	89.7 ± 2.8	0–100	
Wild^b^	21	15.0 ± 0.7	6–24	22	4.0 ± 0.7	1–11	34	45.2 ± 7.5	0–100		19	73.2 ± 8.0	0–100	

a Eggs produced by a laboratory-reared adults.

b Eggs produced by a wild-collected adults.

### Growth

**Fig. 3.**
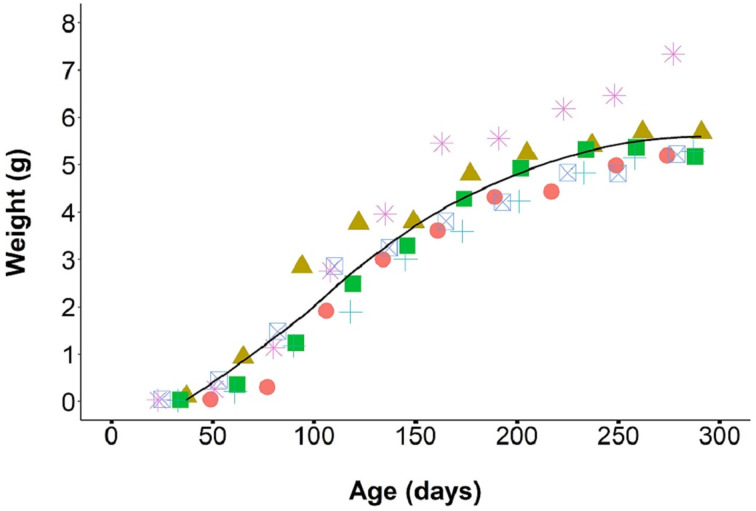
Growth rate of *P. martensi* from neonate to reproductive adult. Different coloured symbols represent separate cohorts (6 cohorts, total 43 individuals).

From August 2019 through April 2020, we monitored the growth of 43 F1 individual laboratory-reared *P. martensi* between 23 and 303 days old. The individuals were housed in six different cohorts, ranging from 3 to 15 individuals and weights were taken every 4 weeks. At 23 days, all juveniles reached at least 0.01 g. Juvenile growth during the first 30 days was relatively slow at 0.002 g day^−1^. At about 30 days of age, the juveniles entered a more rapid growth phase, averaging 0.03 g day^−1^ up to about 200 days of age (Fig. 3). During this phase, food consumption was noticeably higher. It was also at this time that variance in size between siblings became apparent. Size differences between the largest and smallest individual hatched from the same egg cluster ranged from 0.10 to 4.16 g (mean = 1.19), and these size variances were maintained into adulthood. As individuals reached reproductive maturity, food consumption and growth rate began to slow. The mean adult weight at first egg production was 3.96 ± 0.2 g, which was significantly higher than the weight of reproductive wild-collected adults (1.53 ± 0.1 g) (*W* = 0, *P* < 0.001). Survivorship post egg hatch was 86.2 ± 2.9 (median = 100, range = 0–100)% at 30 days (neonates had a mortality rate of about 14%) and 99% thereafter for up to a year.

## Discussion

*Parmarion martensi* laboratory rearing has been documented once before in Taiwan, but with different results than we observed. Liu *et al*. (1997) reared *P. martensi* on cabbage either singly or in pairs at temperatures ranging from 20 to 28°C and recorded longevity, reproduction and hatch rate. Liu *et al*. (1997) measured lifespan averaging 84–139 days as compared to 280–339 days in our study; hatch rates were 74.3–92.9% at the higher temperatures whereas we observed no egg hatch; and they observed self-fertilization and fecundity rates of singly held adults that were similar to those of paired adults (68.6%) whereas we observed little or no fecundity in adults held alone. These discrepancies may suggest that Liu *et al*. (1997) were studying a different species of semi-slug. Identification of *P. martensi* is difficult (Hollingsworth *et al.*, 2007). For example, *P. martensi* is only distinguishable from the widely distributed *Parmarion pusillus* by the relative lengths of the dart sac and dart gland (Schilthuizen and Liew, 2008).

### Behaviour

Since we made all observations during daylight hours only, documentation of all behaviours was not possible. Nonetheless, resting behaviours were observed, and evidence of nocturnal activities was noted during the frequent daylight inspections. During daylight hours, individuals most commonly had congregated under one plastic pot in a tight group, often maximizing bodily contact. Although many slug species are reported to aggregate when conditions are dry, thus reducing evaporation (Cook, 1981), this behaviour was consistent even though humidity was maintained at >95% for *P. martensi* in the lab, and even when other similar shelters were available in their containers. This behaviour did not change when individuals from an un-related cohort were placed in a container together.

We frequently found feces distributed throughout the rearing containers and on the lid, confirming that a significant amount of movement occurred during the night. As individuals approached reproductive age, considerable amounts of clear or white slime could be found throughout the rearing containers. In other slug species, this can be attributed to either aggression (Rollo and Wellington, 1979) or reproduction (Nicholas, 1984). However, we never observed any injuries to individuals, even when densities were high (i.e. 15 adults per container), and overall mortality was extremely low. When we found a deceased adult, there was no sign of cannibalism. However, we did observe egg clusters that had been partially eaten by adults and recently hatched neonates consistently fed on their egg cluster as well. Adults would often lose their shells near the end of their lives, but this was not associated with ensuing mortality. From 2 days after hatch through adulthood, we observed the ability for *P. martensi* to slowly rappel themselves down from an object by threads of viscous mucus.

### *Parmarion martensi* in Hawai‘i

*Parmarion martensi* was first documented in Hawai‘i on the island of O‘ahu in 1996 (Cowie, 1997) and shortly thereafter in east Hawai‘i Island in 2004, but may have arrived as early as 1999 (Hollingsworth *et al*., 2007). While there are no abundance or density data for *P. martensi* in Hawai‘i, surveys have found high infection prevalence and high infection parasite loads in individuals collected broadly across east Hawai‘i (Hollingsworth *et al*., 2007; Qvarnstrom *et al*., 2013) and this region has also had the highest number of neuroangiostrongyliasis cases in Hawai‘i (Johnston *et al*., 2019). These high infection levels, in combination with its climbing behaviour and association with human dwellings, indicate this species should be considered a high-risk to humans. One individual collected from east Hawai‘i Island contained >6800 infective third stage *A. cantonensis* larvae (Hollingsworth *et al*., 2013). Field control methods for slugs and snails include minimizing available areas or objects suitable as hiding places or using poisoned baits, such as those containing metaldehyde and iron phosphate (Hollingsworth *et al*., 2013).

*Parmarion martensi* has been found on Hawai‘i produce exported to California, e.g. sweet potatoes and taro leaves (Follett, pers. communication). Fresh produce exported from Hawai‘i to the U.S. mainland that may harbour quarantine insect pests require the application of a postharvest quarantine treatment such as irradiation (Follett, 2014; Barkai-Golan and Follett, 2017). Other invertebrates can also be quarantine pests and may require a disinfestation treatment. The semi-slug *P. martensi* is considered a quarantine pest as it does not occur in the continental United States. Irradiation studies are needed to identify a treatment dose to stop reproduction in *P. martensi* and thereby prevent any invasive establishment.

### Research potential

Although laboratory systems exist on Hawai‘i Island, the majority of research conducted on *A. cantonensis* and its most competent intermediate host relies on wild collections of *P. martensi.* A healthy and parasite-free laboratory colony eliminates uncertainty in the infection status in test subjects and helps reduce the risk of worker exposure; as such, uninfected *P. martensi* can be challenged in a controlled manner with *A. cantonensis* or other pathogenic nematodes at known infection rates. This will help in the evaluation of control strategies such as UV, ozonation and vegetable washes. A parasite-free colony will also facilitate research concerning the molecular and biochemical pathways of *P. martensi* immune reactions with *A. cantonensis*, as host–pathogen interactions may be crucial to the development of drugs, therapies and control strategies for zoonotic parasitic nematodes (Penagos-Tabares *et al*., 2018).

## Supplementary material

For supplementary material accompanying this paper visit https://doi.org/10.1017/S0031182020001353. click here to view supplementary material
